# DNA Repair Gene Mutations as Predictors of Immune Checkpoint Inhibitor Response beyond Tumor Mutation Burden

**DOI:** 10.1016/j.xcrm.2020.100034

**Published:** 2020-06-23

**Authors:** David Hsiehchen, Antony Hsieh, Robert M. Samstein, Tianshi Lu, Muhammad S. Beg, David E. Gerber, Tao Wang, Luc G.T. Morris, Hao Zhu

**Affiliations:** 1 Division of Hematology and Oncology, Department of Internal Medicine, University of Texas Southwestern Medical Center, Dallas, TX 75390, USA; 2 Division of Gastroenterology, Department of Medicine, University of Pennsylvania, Philadelphia, PA 19104, USA; 3 Department of Radiation Oncology, Icahn School of Medicine at Mount Sinai, New York, NY 10029, USA; 4 Precision Immunology Institute at Icahn School of Medicine at Mount Sinai, New York, NY 10029, USA; 5 Quantitative Biomedical Research Center, Department of Population and Data Sciences, University of Texas Southwestern Medical Center, Dallas, TX 75390, USA; 6 Department of Population and Data Sciences, University of Texas Southwestern Medical Center, Dallas, TX 75390, USA; 7 Immunogenomics Precision Oncology Platform, Memorial Sloan Kettering Cancer Center, New York, NY 10065, USA; 8 Human Oncology and Pathogenesis Program, Memorial Sloan Kettering Cancer Center (MSKCC), New York, NY 10065, USA; 9 Children’s Research Institute, Department of Pediatrics, and Department of Internal Medicine, Center for Regenerative Science and Medicine, University of Texas Southwestern Medical Center, Dallas, TX 75390, USA

**Keywords:** immunotherapy, cancer biomarkers, survival, DNA repair

## Abstract

Immune checkpoint inhibitors (ICIs) have revolutionized cancer therapy, but prediction of their benefit is challenging. Neoantigens generated through impaired non-mismatch DNA repair may result in greater ICI activity. By analyzing 1,661 ICI-treated patients, we show that deletions and mutations in nucleotide excision repair (NER) and homologous repair (HR) pathways are predictors of ICI benefit independent of tumor mutation burden and tumor type. NER and HR mutations are also associated with objective response rates to ICIs in esophagogastric and non-small-cell lung cancers. In a cohort of 40,181 unique patients, NER and HR mutations are present in 3.4% and 13.9% of cancers, respectively. These results indicate that NER and HR gene mutations occur in a subpopulation of cancer patients and may aid patient selection for ICI therapy. Assessing NER and HR mutations in the context of other biomarkers may yield powerful predictors of ICI activity across different cancer types.

## Highlights

NER and HR gene mutations predict overall survival benefit with immunotherapy
Predictive value of NER and HR gene mutations is independent of mutation burden
Objective responses to immunotherapy are more frequent in NER and HR mutant cancers
NER and HR gene mutations occur in 3.4% and 13.9% of cancers, respectively

## Graphical Abstract

**Figure:**
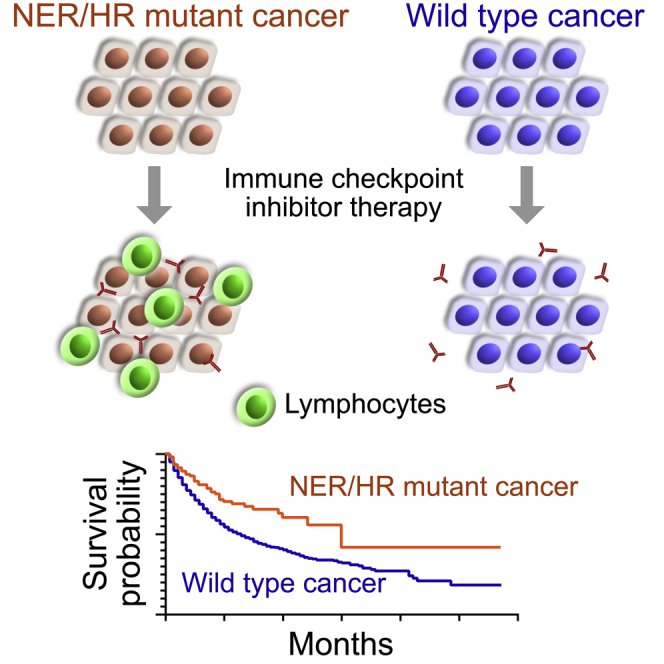

## Teaser

Hsiehchen et al. show that NER and HR gene mutations are associated with longer survival in cancer patients treated with immunotherapy. This association is seen in multiple cancer types and independent of mutation burden. NER and HR gene mutations occur in 3.4% and 13.9% of annotated cancers, respectively.

## Introduction

Targeted manipulation of immune checkpoints, including PD-1 and CTLA-4, yields striking and durable responses in diverse cancer types, but such responses are challenging to predict. While existing biomarkers, including PD-L1 expression and mismatch repair deficiency (MMRd) or microsatellite instability (MSI), are currently used to guide treatment selection, they also have limited predictive and clinical value. Neither high PD-L1 expression, MMRd, or MSI are sufficient to drive immune checkpoint inhibitor (ICI) response, and benefit from ICIs has been observed in a non-trivial portion of biomarker-negative patients.^1^, ^2^, ^3^, ^4^ In particular, while up to 40% of MMRd/MSI cancers may demonstrate a response to ICIs, the prevalence of MMRd/MSI is infrequent across all cancer types, with most cancers having no or rare evidence of MSI.^5^^,^^6^ The lack of more robust clinical tools to guide ICI use has led to a growing number of Food and Drug Administration-approved indications that are independent of biomarkers.^2^^,^^7^ However, overtreatment with ICIs remains a considerable concern, particularly when some patients demonstrate greater benefit from other modalities.^8^ Clearly, improved predictive biomarkers of ICI response are needed to advance precision medicine and improve patient outcomes.

An association between tumor mutation burden (TMB) with ICI response has been observed in different cancer types due to the potential for somatic mutations to encode immunogenic antigens.^9^ High TMB is associated with greater neoantigen loads and immune infiltration, consistent with the importance of neoantigens in immune rejection and ICI efficacy.^3^ Non-MMR DNA repair pathways may increase somatic mutation rates, but their association with ICI response has not been examined systematically.^10^

We hypothesize that mutations in non-MMR DNA repair pathways may engender neoantigens and predict cancers that respond to ICIs. To test this hypothesis, we investigated genetic datasets paired with clinical data from ICI-treated patients who were evaluated by the MSK-IMPACT targeted next-generation sequencing gene panel (Table 1).^9^ This cohort included 1,661 patients with metastatic or unresectable cancers treated with antibodies targeting CTLA-4, PD-1/PD-L1, or both. We examined mutations across multiple DNA repair pathways, including base excision repair (BER), MMR, nucleotide excision repair (NER), Fanconi anemia repair (FA), homologous recombination (HR), and DNA checkpoints (DNACHKs), and discovered that only NER and HR gene mutations were associated with longer overall survival in ICI-treated patients independent of tumor type and TMB. We also examined subgroups of ICI-treated patients with esophagogastric adenocarcinoma and non-small-cell lung cancer (NSCLC) who had objective response rate data available, and we show that cancers with NER or HR gene mutations were enriched in cancers that exhibited a complete or partial response to ICIs. By profiling 40,181 annotated cancers, we found that 3.4% and 13.9% of cancers harbored NER and HR mutations, respectively. Collectively, these findings suggest that NER and HR gene mutations may represent predictive biomarkers of ICI sensitivity and may broaden the clinical usage of ICIs to more patients and cancer types.

**Table 1:** DNA Repair Genes in the MSK-IMPACT Sequencing Panel

Base Excision Repair	Mismatch Repair	Nucleotide Excision Repair	Fanconi Anemia Repair	Homologous Recombination	DNA Checkpoints
MUTYH PARP1 NTHL1	MSH2 MSH3 MSH6 MLH1 PMS2 PMS1	ERCC3 ERCC2 ERCC5 ERCC4	FANCA FANCC BRCA2 BRIP1 PALB2 RAD51C SLX4	RAD51 RAD51B RAD51D XRCC2 RAD52 RAD54L RAD50 MRE11 NBN ARID1A BLM BRCA2	DNACHK MDC1 ATM ATR BRCA1 CHEK1 CHEK2 TP53BP1

## Results

**Figure 1:**
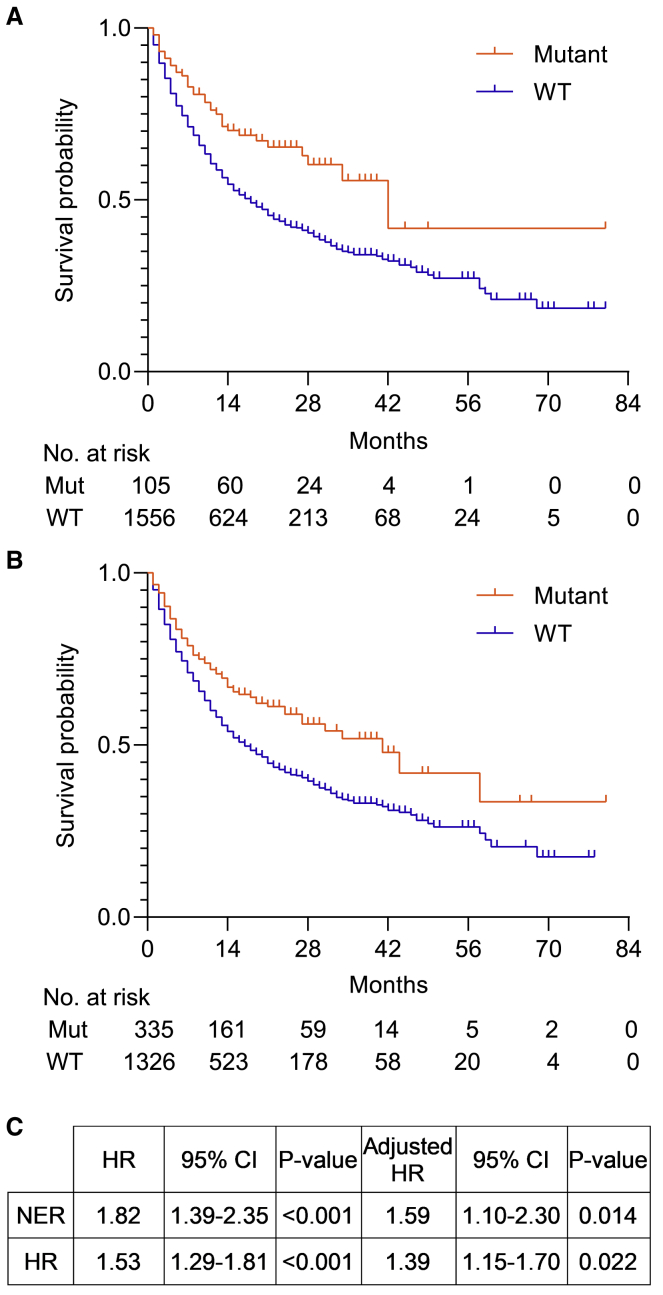
DNA Repair Gene Mutations Are Associated with Longer Overall Survival in ICI-Treated Patients (A) Unadjusted Kaplan-Meier survival curves for NER mutant (Mut) and wild-type (WT) patients treated with ICIs. (B) Unadjusted Kaplan-Meier survival curves for HR Mut and WT patients treated with ICIs. (C) Unadjusted and adjusted hazard ratios (HRs) for overall survival with 95% confidence intervals (95% CI) associated with NER and HR mutations. The adjusted Cox regression model included TMB, type of ICI administered, and tumor type.

Among 1,661 ICI-treated patients, mutations in NER (median overall survival, 42 months vs. 18 months; χ^2^c = c12.7, p < 0.001) and HR (median overall survival, 41 months vs. 16 months; χ^2^c = c20.3, p < 0.001) were associated with significantly longer overall survival compared to the wild-type population (Figure 1).^9^ Adjustment for high TMB, type of ICI administered, and tumor type using a Cox regression model showed that NER (p = 0.014; hazard ratio, 1.59; 95% confidence interval [CI], 1.10–2.30) and HR (p = 0.022; hazard ratio, 1.39; 95% CI, 1.15–1.70) mutations remained significantly associated with improved outcomes. BER, MMR, FA, and DNACHK mutations as well as mutations in *POLE/POLD1* were only significantly associated with longer overall survival in unadjusted analyses, but not after adjustment for TMB or tumor type.

ARID1A was included in the HR gene panel because it localizes to sites of double-strand breaks and is required for efficient HR, suggesting that it is directly involved in HR-dependent DNA repair.^11^, ^12^, ^13^, ^14^ Multiple studies have also demonstrated that ARID1A knockdown or knockout sensitizes cells to PARP inhibitors, which is reminiscent of the synthetic lethal relationship between BRCA1/2 and PARP1.^13^^,^^14^ These findings have led to the inclusion of *ARID1A* in HR gene panels of ongoing clinical studies to stratify patients (ClinicalTrials.gov: NCT04042831, NCT03207347, and NCT03209401). However, given that ARID1A has also been implicated in other cellular processes, we assessed whether the clinical benefit of HR gene mutations among ICI-treated patients was due to the inclusion of *ARID1A*. Removal of *ARID1A* from the HR gene panel showed that mutations in the remaining HR genes were still associated with a longer overall survival (median overall survival, 41 months vs. 17 months; χ^2^c = c15.1, p < 0.001). In addition, the HR gene panel without ARID1A remained independently associated with longer overall survival after adjusting for high TMB, type of ICI administered, and tumor type (p = 0.015; hazard ratio, 1.36; 95% CI, 1.06–1.74). This suggests that canonical HR genes are, indeed, predictive of ICI benefit, and for subsequent analyses, we have retained ARID1A in the HR gene panel.

The association between NER and HR gene mutations and longer overall survival may be due to individual or aggregate effects of gene mutations. However, the frequency of individual gene mutations in NER and HR was generally rare, with the exception of *ARID1A* (11.4%) and *BRCA2* (5.6%). Mutations in *ARID1A* (hazard ratio, 1.41; 95% CI, 1.11–1.79) and *BRCA2* (hazard ratio, 1.48; 95% CI, 1.07–2.05) were individually associated with a hazard ratio greater than 1. Less common genes with at least 1% incidence in the ICI-treated cohort were also generally associated with hazard ratios greater than 1, including *RAD50* (hazard ratio, 2.40; 95% CI, 1.14–5.06), *RAD51B* (hazard ratio, 1.93; 95% CI, 0.86–4.32), *MRE11* (hazard ratio, 2.27; 95% CI, 1.08–4.78), *NBN* (hazard ratio, 2.18; 95% CI, 1.09–4.38), *BLM* (hazard ratio, 1.25; 95% CI, 0.76–2.05), *ERCC2* (hazard ratio, 1.59; 95% CI, 0.92–2.75), *ERCC3* (hazard ratio, 2.22; 95% CI, 0.92–5.35), *ERCC4* (hazard ratio, 2.07; 95% CI, 1.08–4.00), and *ERCC5* (hazard ratio, 1.85; 95% CI, 1.02–3.36).

**Figure 2:**
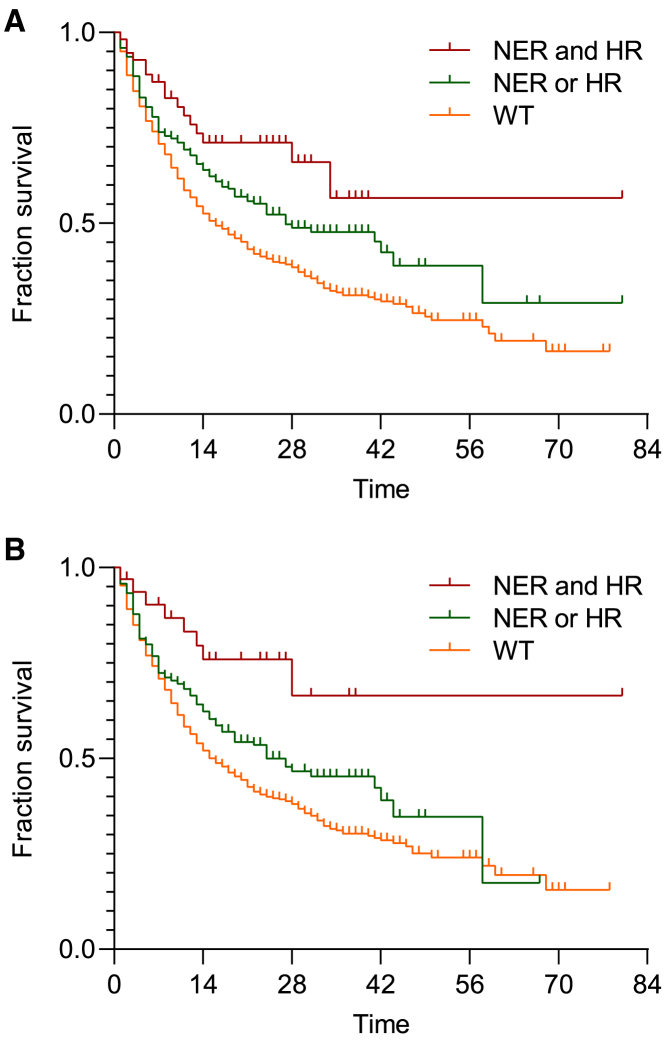
NER and HR Mutations Are Independent and Additive Predictors of ICI Response (A) Kaplan-Meier survival curves for ICI-treated patients with no mutations (WT), NER or HR mutations, or NER and HR mutations. (B) Kaplan-Meier survival curves for non-MMRd ICI patients with no mutations (WT), NER or HR mutations, or NER and HR mutations.

A Cox regression model including NER gene mutations (p = 0.003; hazard ratio, 1.68; 95% CI, 1.19–2.36) and HR gene mutations (p < 0.001; hazard ratio, 1.44; 95% CI, 1.19–1.74) showed that mutations of distinct DNA repair pathways were predictors of ICI benefit independent of each other. Adjusting for high TMB showed that NER (p = 0.008; hazard ratio, 1.59; 95% CI, 1.13–2.25) and HR (p = 0.002; hazard ratio, 1.36; 95% CI, 1.11–1.66) gene mutations remained significantly and independently associated with longer overall survival. To determine whether mutations in distinct DNA repair pathways may have an additive impact, we stratified patients who had mutations in both NER and HR, in NER or HR, or neither. The median overall survival was 16 months for wild-type patients, 27 months for patients with either NER or HR gene mutations, and not reached for patients with NER and HR gene mutations (χ^2^c = c23.5, p < 0.001) (Figure 2A). Given that MMRd may result in the accumulation of mutations in multiple DNA repair pathways, we performed similar analyses after excluding MMRd cancers. Among the remaining 1,516 patients, the median overall survival was 16 months for wild-type patients, 24 months for patients with either NER or HR gene mutations, and not reached for patients with both NER and HR gene mutations (χ^2^c = c17.4, p < 0.001) (Figure 2B).

To exclude the possibility that NER and HR gene mutations are prognostic markers regardless of ICI exposure, we examined cancers previously characterized by MSK-IMPACT that were not treated with ICIs.^15^ NER gene mutations were not significantly associated with overall survival, while HR gene mutations were associated with worse overall survival (p = 0.003; hazard ratio, 0.86; 95% CI, 0.78–0.95). Individual analysis of TCGA datasets of the most common cancer types represented in the ICI-treated cohort—including NSCLC (n = 586), renal cell carcinoma (n = 538), esophageal and gastric cancer (n = 664), colorectal cancer (n = 640), and glioblastoma (n = 604)—showed no significant associations between NER and HR gene mutations with overall survival.

**Figure 3:**
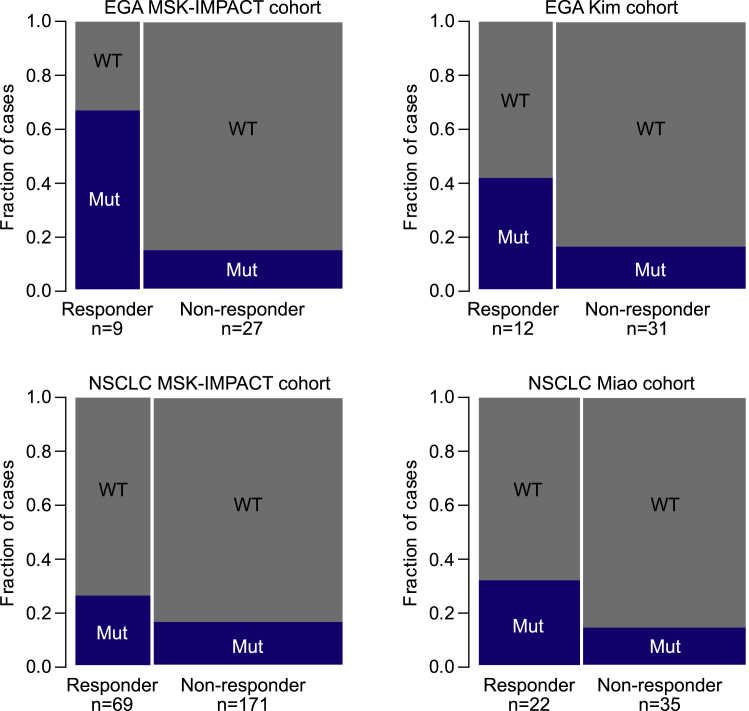
Association between Objective Response Rates to ICIs and NER and HR Mutations The proportions of NER and HR Mut or WT cancers are shown for patients who had a complete or partial response (Responder) and for patients who did not (non-responder) in separate cohorts of patients with esophagogastric adenocarcinoma (EGA) or non-small-cell lung cancer (NSCLC).

Objective response rates from the ICI-treated MSK-IMPACT cohort were available for patients with esophagogastric adenocarcinoma (EGA) and NSCLC. We thus assessed whether NER and HR gene mutations were more prevalent in cancers that had either a complete response or a partial response to ICIs (Figure 3). Indeed, there was a statistically significant greater proportion of cancers with NER and HR gene mutations among patients who had an objective response compared to those who did not have an objective response in both EGA (*Z* score 3.01, p = 0.001) and NSCLC cohorts (*Z* score 1.73, p = 0.041). Given the small sample sizes and low frequency of NER and HR gene mutations, NER and HR mutations were aggregated in these analyses. To validate these results, we performed identical analyses on additional cohorts of ICI-treated patients with EGA and NSCLC from separate studies.^16^^,^^17^ Similar to the MSK-IMPACT cohorts, we found that the fraction of patients with NER and HR gene mutations in responders compared to non-responders was significantly greater in EGA patients (*Z* score = 1.778, p = 0.037) and neared a significant difference in NSCLC patients (*Z* score = 1.580, p = 0.057).

**Figure 4:**
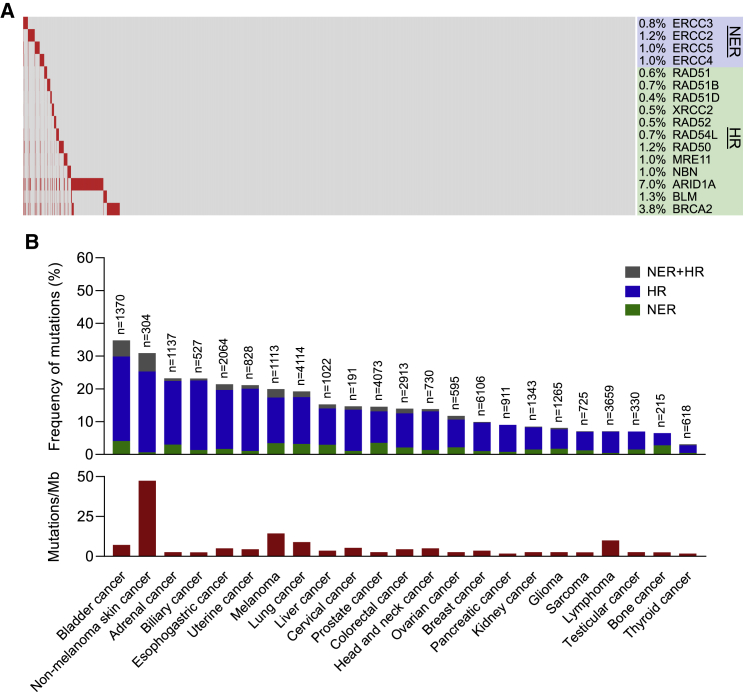
Landscape of NER and HR Mutations across Different Cancers (A) Waterfall plot of NER and HR mutations across 40,181 unique cancers. (B) Frequency of NER and HR mutations and median TMB by cancer type.

Among 40,181 unique cancers whose genomes have been previously characterized, 3.4% and 13.9%, cancers had NER and HR gene mutations, respectively (Figure 4A). The frequency of NER and HR gene mutations varied across cancer types, ranging from 34.8% in non-melanoma skin cancers to 3.1% in thyroid cancers (Figure 4B). NER gene mutations were most frequent in bladder cancer (4.1%), prostate cancer (3.5%), and melanoma (3.4%). HR gene mutations were most frequent in bladder cancer (25.7%), non-melanoma skin cancer (24.7%), and biliary cancer (21.3%). There was no correlation between the frequency of NER and HR gene mutations with median TMB values assessed in a prior study across different cancer types (Figure 4B).^18^

## Discussion

Copy number alterations, gene expression signatures, and driver gene mutations have been linked to ICI response or resistance, although these mechanisms have principally been described in a few cancer types, and their relation to TMB has not been fully explored.^17^^,^^19^, ^20^, ^21^, ^22^, ^23^, ^24^, ^25^ Our study indicates that NER and HR gene mutations may be predictive of ICI response across different cancer histologies and independent of TMB. In support of this, genes within our HR gene panel, including *ARID1A* and *BRCA2*, have been associated with ICI sensitivity.^26^^,^^27^ In particular, inactivation of *ARID1A in vivo* increases tumor-infiltrating lymphocytes and sensitivity to anti-PD-L1 antibody therapy in ovarian cancer, while *BRCA2* mutations are enriched in melanomas responsive to ICIs.^26^^,^^27^ In addition, a smaller study of urothelial carcinoma previously demonstrated a higher response rate of ICIs in cancers with DNA damage response gene mutations, including those of unknown significance.^28^ The mechanism of how DNA repair defects may enhance ICI activity beyond the accumulation of somatic mutations remains to be elucidated, though impaired DNA repair mechanisms may modulate innate immune processes such as the cGAS-STING pathway in cancer cells.^29^ Disruption of NER and HR may also induce specific mutational signatures or other cellular effects that enhance ICI activity.^27^

There are several limitations to this study. First, whether nonsynonymous mutations observed across DNA repair genes result in functional defects that cause impaired DNA repair was not tested. Nonetheless, saturation mutation studies of notable tumor suppressor genes including *BRCA1*, *TP53*, and *PTEN* suggest that missense mutations in tumor suppressor gene have a substantial probability of altering protein function.^30^, ^31^, ^32^ Future studies examining the functional consequences of mutations in HR and NER gene panels are needed to understand the mechanism underlying their association with ICI benefit. Second, our analysis of response rates was limited to two cancer types given the current availability of data. Notably, objective responses may not correlate with long-term clinical benefit; thus, NER and HR gene mutations may not necessarily predict objective response rates in patients who may still benefit from ICIs.^33^^,^^34^ In addition, the association between NER and HR gene mutations with response rates in additional cancer types remains to examined. Third, the ICI-treated cohort does not incorporate cancer types where ICIs are not used in routine patient care or have not been extensively tested in clinical trials. Thus, whether NER and HR gene mutations may predict ICI benefit in cancer types not examined in this study remains to be investigated.

While NER and HR gene mutations were observed in many cancer types, including those with relatively low median TMBs, it remains to be determined whether ICI sensitivity correlates with DNA repair gene mutation frequency, and caution is needed in extrapolating these findings. Based on the synthetic lethality between PARP1 and HR deficiency, the ATLAS study—a phase II, open-label study of rucaparib, a PARP1 inhibitor, in unselected patients with locally advanced or metastatic urothelial carcinoma—was recently terminated due to a lack of objective responses, despite the relatively high prevalence of HR mutations in urothelial cancers. However, PARP1 inhibitor sensitivity may be a poor surrogate of HR deficiency given the multitude of parylation substrates and non-enzymatic functions of PARP1.

Our results indicate that NER and HR gene mutations may aid patient selection for ICI therapy, although prospective validation is necessary. Importantly, NER gene mutations are currently not actionable findings, but our results suggest that they may be amenable to ICI therapy. Our data support the testing of ICIs in non-MMRd NER and HR mutant cancers agnostic of tumor histology. NER and HR gene mutations likely account for one of many aspects of tumor biology that may contribute to ICI sensitivity. Thus, further exploration and refinement of NER and HR gene mutation panels and testing of their use in combination with existing and emerging biomarkers, including algorithms that assess the clonality and immunogenicity of neoantigens, may yield more powerful predictors of ICI activity across many cancer types.^35^

## STAR★Methods

### Key Resources Table

**Table:** 

REAGENT or RESOURCE	SOURCE	IDENTIFIER
**Deposited Data**		

Mutation calls and clinical subject data for ICI treated cohort	https://www.cbioportal.org	tmb_mskcc_2018

### Resource Availability

#### Lead Contact

Further information and requests for resources and reagents should be directed to and will be fulfilled by the Lead Contact, David Hsiehchen (gbtwnow@gmail.com).

#### Materials Availability

This study did not generate new unique reagents.

#### Data and Code Availability

This study did not generate any unique datasets or code.

### Experimental Model and Subject Details

The ICI-treated cohort includes 1,661 patients with metastatic or unresectable cancers at Memorial Sloan Kettering Cancer Center that were assessed by targeted next generation sequencing (NGS).^9^ This cohort included ten cancer types: bladder, breast, colorectal, esophagogastric, glioma, head and neck, melanoma, non-small cell lung, renal cell, and unknown primary cancer. Mean age was 61 years (standard deviation 14 years) and 62.2% of patients were male. ICI treatment could have been administered as first or subsequent line of therapy and included antibodies targeting CTLA-4 (9%), PD-1/PD-L1 (76%), or both (16%).

### Method Details

MSK-IMPACT is a hybridization capture-based NGS panel analyzing formalin-fixed, paraffin-embedded tumors and matched peripheral blood for 410 or 468 cancer associated genes including protein-coding mutations, copy number alterations, and select promoter mutations and structural rearrangements.^15^^,^^36^ Bioinformatic analysis of sequencing data was performed as previously described in prior MSK-IMPACT studies.^9^^,^^15^^,^^36^ Genomic DNA is extracted from tissue specimens with at least 10% tumor cells, with preferably greater than 20% viable tumor. Sequence libraries are prepared using KAPA Biosystems Library Preparation Reagents and captured using custom designed biotinylated probes (NimbleGen) for targeted sequencing of all protein-coding exons and selected introns of oncogenes and tumor suppressor genes. Massive parallel sequencing of pooled libraries is performed on Illumina HiSeq 2500

Instruments. Mutation calling pipeline includes several filters to eliminate artifacts, low quality sequencing data, and germline mutations.

Genes included in the MSK-IMPACT targeted next-generation sequencing panel were classified into different DNA repair pathways as shown in Table 1 which include base excision repair, MMR, nucleotide excision repair, Fanconi anemia repair, homologous recombination, and DNA checkpoints.^37^^,^^38^ Nonsynonymous mutations including missense, nonsense, frameshift, and splice site changes of genes were considered as well as biallelic deletions.

### Quantification and Statistical Analysis

Overall survival was determined from the date of first ICI treatment to time of death or most recent follow-up with a median follow-up of 19 months. Kaplan-Meier survival curves were compared using log-rank tests. Adjusted hazard ratios were determined using Cox regression models including TMB, type of ICI administered, and tumor type as covariates. We defined TMB-high cancers as those within the highest mutation load quintile for each histology which was previously demonstrated to be a robust predictor of ICI response in the MSK-IMPACT cohort. This percentile cut-off is advantageous given the varying mutation load across different cancer types and may explain the inconsistent results in other studies assessing TMB as a predictor of ICI benefit using absolute cut-offs. A binary indicator variable was included in Cox regression models to distinguish cancers with the highest 20% TMB per histology. The two-sample z test of proportions was used to test for statistical differences in the proportion of patients with NER and HR mutations among ICI-treated patient responders and non-responders.

SPSS Statistics 23 (IBM) and GraphPad Prism 8 were used to generate Kaplan-Meier curves and to perform Cox regression analyses. The proportional hazard assumption was tested by including a time-dependent covariate to each model and results were only reported if the covariate was not significantly associated with outcome indicating that the assumption was not violated.
